# Supplementary material for: Large Cancer Pedigree Involving Multiple Cancer Genes including Likely Digenic MSH2 and MSH6 Lynch Syndrome (LS) and an Instance of Recombinational Rescue from LS
Source: Cancers (Basel). 2022 Dec 30;15(1):228. doi: 10.3390/cancers15010228 (PMC9818763; doi:10.3390/cancers15010228)
Supplement: Supplementary file 1 [file cancers-15-00228-s001.zip › cancers-2071483-supplementary.pdf]

Supplementary Materials and Methods

Samples received

The samples received from members of Family C (Figure 1) are listed below.

The following family members provided a saliva sample in an Oragene DNA Collection Kit for DNA analysis, as follows:

II.12, II.15, III.1, III.2, III.3, III.5, III.6, III.9, III.10, III.11, III.15, III.18, III.19, III.20, III.23, III.24, III.25, III.26, III.27, III.28, III.29, III.30, III.31, III.32, III.34, III.35, III.36, III.39, III.40, III.41, IV.1, IV.2, IV.3, IV.4, IV.5, IV.6, IV.7, IV.8, IV.9, IV.10, IV.11, IV.12, IV.13, IV.14, IV.15, IV.16, IV.17, IV.18, IV.19, IV.20, IV.21, IV.22, IV.23.

All family members were invited to provide an additional saliva sample in an Oragene RNA collection vial. A subset of 16 family members provided an additional saliva sample. Three (IV.13, IV.14, IV.15) international samples were degraded on arrival. One (II.15) leaked in transit due to a loose lid. RNA analyses on saliva were completed on 12 family members, including III.1, III.3, III.5, III.9, III.11, III.15, III.18, III.19, IV.1, IV.2, IV.11, IV.14 (Figure 4, Figure 5, Supplementary Figure 4, Supplementary Figure 5).

The following family members provided other sample types, as follows:

III.20 (Proposita, carrier of both *MSH2* and *MSH6* variants): Blood (two K_2EDTA tubes). One tubes was used to isolate peripheral blood leukocytes (PBLs) for genomic DNA. The other tubes was used to isolate peripheral blood mononuclear cells (PBMCs) for RNA analyses (Figure 4A, B).

III.19 (Proposita sister, carrier of both *MSH2* and *MSH6* variants): In addition to saliva (DNA and RNA), III.19 also provided blood samples on three separate occasions (over a period of 9 years). The first blood draw (one K_2EDTA tube) was used for next-generation sequencing and array comparative genomic hybridization (aCGH) of PBL at an independent CLIA-approved laboratory (Mayo Clinic) to verify carriage of the *MSH2* and *MSH6* variants. The second blood draw was used for RNA analyses of *MSH2* and *MSH6* in whole blood (from one Zymo DNA/RNA Shield tube) and in PBMCs (from one K_2EDTA tube) (Figures 4C, 5, and 6). The third blood draw (one ACD collection tube) was used for genomic DNA analysis by linked-read whole genome sequencing.

III.3 (carrier of *MSH2* variant): In addition to initial samples of saliva (DNA and RNA), III.3 provided a second set of samples, including blood (one K_2EDTA tube) from which PBMCs were isolated, Saliva (RNA tube), and Hair follicles, all collected on the same day approximately six months later. These were used for *MSH2* RNA analyses (Figure 4D, E), and *MSH6* RNA analyses (as a non-carrier, Supplementary Figure 6).

IV.14 (carrier of the *MSH2* variant): The saliva RNA was degraded on arrival. Hair follicles provided were used for RNA analyses (Supplementary Figures 4 and 5).

III.13 (Proband, deceased. Carrier of both *MSH2* and *MSH6* variants): Formalin-fixed paraffin embedded (FFPE) tissue block of a rectal cancer biopsy was retrieved from the treating hospital's Pathology Archive.

III.14 (Proband's brother, deceased. Carrier of both *MSH2* and *MSH6* variants): FFPE tissue block of colon cancer was retrieved from the treating hospital's Pathology Department.

III.15 (Proband's living brother. Carrier of *MSH6* variant): FFPE block containing a fragment of a 3mm colonic tubular adenoma.

III.18 (Proband's living brother. Carrier of *MSH6* variant): One FFPE block containing a fragment of a 2-3mm hyperplastic polyp. A second FFPE block containing a fragment of a 2-3mm colonic tubular adenoma.

A healthy control (HC) subject, who is a female non-carrier of either *MSH2* or *MSH6* variant proven by targeted sequencing and the Invitae 134 multi-gene panel test, provided the following samples for RNA analyses: Whole blood (Zymo DNA/RNA Shield), PBMCs (K_2EDTA), EBV-transformed lymphoblastoid cell line, saliva (RNA), hair follicles.

Table S1. Primers and PCR amplification conditions used for targeted testing of genomic DNA for the germline sequence variants *MSH2* c.2006G>T and *MSH6* c.3936_4001+8dup.

Gene	Forward primer sequence (5' → 3')	Reverse primer sequence (5' → 3')	Tm ^{0}C	Amplicon size (expected), bp
MSH2	CGCGAT-TAATCATCAGTG	GGACAGAGACATACATTTC-TATC	50	353
MSH6	CTGTGCGCCTAG-GACATAT	TCATAGTGCATCATCC CTTC	60	399

PCRs were performed on 20-50ng genomic DNA as template. PCR protocol was the same for both assays: An initial denaturation cycle at 95^{0}C, followed by 35 cycles of denaturation (30 seconds at 95°C), annealing at relevant annealing temperature (Tm) (40 seconds), and extension (30 seconds at 72°C). A final extension was performed for 10 minutes at 72 °C. The *MSH6* c.3936_4001+8dup allele generated a PCR product 74 bp larger than the expected product size (473 bp).

Table S2. Primers and PCR amplification conditions used for reverse-transcriptase PCR of cDNA templates to determine the effects on splicing of germline sequence variants *MSH2* c.2006G>T and *MSH6* c.3936_4001+8dup.

cDNA	Forward primer sequence (5' → 3')	Reverse primer sequence (5' → 3')	Tm, ⁰C	Normal amplicon size, bp
MSH2	12F: TGCTGTT-GTCAGCTTTGCTC	13R: TTCCAACATTTCAGCCATGA<br>14R: GGTCTCTTCAG-TGGTGAGTGC	58	385<br>622
MSH6	8F: TTTGATGGGACGG-CAATAGCA<br>3F: GTTCAAAATCAAAGGAAGCCC	10R: TATGGACAGCTTCAG-CATCTACAG	60<br>52	391<br>3,671

Each PCR reaction was performed using 2ul (~200ng) cDNA template. For *MSH2*, the same forward primers in exon 12 was used with either reverse primer in exon 13 or 14. PCR protocol was the same for both assays: An initial denaturation cycle at 95⁰C, followed by 35 cycles of denaturation (30 seconds at 95°C), annealing at relevant annealing temperature (Tm) (40 seconds), and extension (30 seconds at 72°C). A final extension was performed for 10 minutes at 72 °C. The expected size of the normal transcript is shown. For *MSH2*, the exon-13 skipped product for the 12F-14R transcript was 417 bp. For *MSH6*, the exon-9 skipped product for the 8F-10R transcript was 191 bp.

Table S3. Locations and sequences of guide RNAs (gRNAs) and binding sites for targeted Chromium 10X linked-read sequencing across a 0.3Mb region of Chr2p21 (chr2:47307193-47598910). .

Target ID	Chr	gRNA binding start	gRNA binding stop	Target orientation	Sequence
upstream-1	chr2	47525924	47525943	sense	CCUAAUAGGUGACGCAGAGU
upstream-2	chr2	47530883	47530902	antisense	CACUUUACAUCAGCCCCCUG
upstream-3	chr2	47534316	47534335	sense	GCCCCUGAUUAACACCAGUG
downstream-1	chr2	47826046	47826065	antisense	UUCCCAUACCUGCUGACGGG
downstream-2	chr2	47831281	47831300	antisense	GAGGCGGUGAUUCCACACCA
downstream-3	chr2	47835792	47835811	antisense	CAACUCAUUCAUACGAGGGG

CLIA-approved external genetic testing for hereditary cancer risk

The contemporary 134-gene multicancer gene panel test was conducted on DNA from fresh samples of saliva by Invitae. The 134 cancer gene panel consists of 83 well-established hereditary cancer-causing genes, plus an additional 51 genes with cancer associations.

The following genes were evaluated for sequence changes and exonic deletions/duplications by next generation sequencing:

ABRAXAS1, AKT1, ALK, APC, ATM, AXIN2, BAP1, BARD1, BLM, BMPR1A, BRCA1, BRCA2, BRIP1, BUB1B, CASR, CDC73, CDH1, CDK4, CDKN1B, CDKN1C, CDKN2A (p14ARF), CDKN2A (p16INK4a), CEBPA, CEP57, CFTR, CHEK2, CTC1, CTNNA1, CTRC, DICER1, DIS3L2, DKC1, EGLN1, ENG, EPCAM, ERCC4, EZH2, FANCA, FANCB, FANCC, FANCD2*, FANCE, FANCF, FANCG, FANCI, FANCL, FANCM, FH, FLCN, GALNT12, GATA1, GATA2, GPC3, GREM1*, HRAS, KIF1B, KIT, MAX, MC1R, MDM2, MEN1, MET, MLH1, MLH3, MRE11, MSH2, MSH3, MSH6, MUTYH, NBN, NF1, NF2, NHP2, NOP10, PALB2, PALLD, PDGFRA, PHOX2B*, PIK3CA, PMS2, POLD1, POLE, POT1, PRKAR1A, PRSS1, PTCH1, PTCH2, PTEN, RAD50, RAD51C, RAD51D, RB1, RECQL4, RET, RINT1, RPL11, RPL26, RPL35A, RPL5, RPS10, RPS19, RPS20, RPS24, RPS26, RPS7, RUNX1, SDHAF2, SDHB, SDHC, SDHD, SLX4, SMAD4, SMARCA4, SMARCB1, SMARCE1, SPINK1, SPRED1, STK11, SUFU, TERC, TERT, TINF2, TMEM127, TP53, TSC1, TSC2, VHL, WRN*, WT1, XRCC2*

The following genes were evaluated only for specific target sequences in which recurrent cancer-associated variants have been identified: *EGFR*, HOXB13*, MITF*, NTHL1*, SDHA*

*Indicates limited testing, as detailed below:

HOXB13: c.251G>A, p.Gly84Glu variant only. *EPCAM*: Deletion/duplication testing only (NM_002354.2). *GREM1*: Promoter region deletion/duplication testing only. *PHOX2B*: Alanine repeat numbers for the commonly-expanded region in exon 3 are not determined. *MITF*: c.952G>A, p.Glu318Lys variant only. *FANCD2*: Deletion/duplication analysis is not offered for exons 14-17 (NM_033084.3). *NTHL1*: Deletion/duplication analysis is not offered for this gene (NM_002528.6). WRN: Deletion/duplication analysis is not offered for exons 10 or 11 (NM_000553.4). *EGFR*: c.2369C>T (p.Thr790Met), c.2327G>A (p.Arg776His), c.2527G>A (p.Val843Ile) variants only.

As described by Invitae's specifications for this panel, genomic DNA is first enriched for the target sequences by hybridization then sequenced using Illumina technology. All targeted regions are sequenced to ≥50x depth. Reads are aligned to the GRCh37 (hg19) human genome reference sequence, and changes identified with reference to a clinically relevant gene transcript. Deletions/duplications are called using a copy number algorithm. Clinically actionable variants identified are then verified by an orthogonal method, such as MLPA, Sanger sequencing, or long-range PCR, depending on the nature of the finding.

Supplementary Results

Table S4. Genotypes and haplotypes for family members III.19 (proband/proposita's sister) and her eldest son IV.11 obtained by 10X Chromium targeted sequencing and linked-read whole genome sequencing.

#chrom	pos	allele_1_III.19	allele_2_III.19	allele_1_IV.11	allele_2_IV.11	base_1_III.19	base_2_III.19	base_1_IV.11	base_2_IV.11	phase_data
chr2	47311338	0	1	0	0	A	G	A	A	targeted
chr2	47321290	0	1	0	0	C	T	C	C	targeted
chr2	47322618	0	1	0	0	T	G	T	T	targeted
chr2	47322624	0	1	0	0	G	T	G	G	targeted
chr2	47324375	0	1	0	0	C	A	C	C	targeted
chr2	47324828	1	1	0	1	C	C	G	C	targeted
chr2	47331974	1	0	0	1	G	A	A	G	targeted
chr2	47338656	0	1	0	0	T	G	T	T	targeted
chr2	47339527	0	1	0	0	A	G	A	A	targeted
chr2	47343924	0	1	0	0	C	T	C	C	targeted
chr2	47344095	1	1	0	1	C	C	T	C	targeted
chr2	47344113	1	1	0	1	T	T	A	T	targeted
chr2	47344167	1	1	0	1	A	A	G	A	targeted
chr2	47345697	0	0	1	0	G	G	A	G	targeted
chr2	47346929	0	0	1	0	C	C	T	C	targeted
chr2	47348110	0	0	1	0	C	C	T	C	targeted

chr2	47348413	0	0	1	0	G	G	T	G	targeted
chr2	47348474	1	1	0	1	A	A	G	A	targeted
chr2	47348613	0	0	1	0	A	A	G	A	targeted
chr2	47350817	0	0	1	0	T	T	A	T	targeted
chr2	47350888	0	0	1	0	G	G	A	G	targeted
chr2	47350891	0	0	1	0	C	C	G	C	targeted
chr2	47351329	0	0	1	0	C	C	G	C	targeted
chr2	47351545	0	0	1	0	G	G	A	G	targeted
chr2	47355562	0	0	1	0	A	A	T	A	targeted
chr2	47356505	0	0	1	0	A	A	G	A	targeted
chr2	47357917	1	0	0	1	C	G	G	C	targeted
chr2	47359998	0	0	1	0	G	G	A	G	targeted
chr2	47362727	0	0	1	0	A	A	G	A	targeted
chr2	47362895	0	0	1	0	C	C	A	C	targeted
chr2	47362931	0	0	1	0	T	T	C	T	targeted
chr2	47363136	0	0	1	0	T	T	C	T	targeted
chr2	47363861	0	0	1	0	C	C	G	C	targeted
chr2	47364384	0	0	1	0	G	G	T	G	targeted
chr2	47365955	0	0	1	0	C	C	T	C	targeted

chr2	47366385	0	0	1	0	G	G	A	G	targeted
chr2	47368571	0	0	1	0	C	C	G	C	targeted
chr2	47372144	0	0	1	0	T	T	C	T	targeted
chr2	47372415	0	0	1	0	G	G	A	G	targeted
chr2	47373967	0	0	1	0	T	T	C	T	targeted
chr2	47375039	0	0	1	0	A	A	T	A	targeted
chr2	47376352	0	0	1	0	A	A	G	A	targeted
chr2	47376521	0	0	1	0	A	A	G	A	targeted
chr2	47379635	0	0	1	0	A	A	G	A	targeted
chr2	47380876	0	0	1	0	A	A	G	A	targeted
chr2	47381993	0	0	1	0	C	C	T	C	targeted
chr2	47383569	0	0	1	0	C	C	G	C	targeted
chr2	47384029	0	0	1	0	G	G	T	G	targeted
chr2	47384482	0	0	1	0	C	C	T	C	targeted
chr2	47385085	0	0	1	0	C	C	T	C	targeted
chr2	47385739	0	0	1	0	G	G	A	G	targeted
chr2	47386731	0	0	1	0	T	T	C	T	targeted
chr2	47387602	0	0	1	0	G	G	T	G	targeted
chr2	47388003	0	0	1	0	C	C	G	C	targeted

chr2	47391539	0	0	1	0	C	C	T	C	targeted
chr2	47391557	0	0	1	0	G	G	C	G	targeted
chr2	47391941	0	0	1	0	T	T	C	T	targeted
chr2	47392501	0	0	1	0	G	G	A	G	targeted
chr2	47394964	1	1	0	1	T	T	C	T	targeted
chr2	47396015	0	0	1	0	G	G	T	G	targeted
chr2	47400522	0	0	1	0	G	G	A	G	targeted
chr2	47400556	0	0	1	0	T	T	C	T	targeted
chr2	47401685	0	0	1	0	C	C	G	C	targeted
chr2	47402465	0	0	1	0	T	T	C	T	targeted
chr2	47403074	0	0	1	0	T	T	C	T	targeted
chr2	47403411	0	0	1	0	C	C	G	C	targeted
chr2	47403500	0	0	1	0	T	T	C	T	targeted
chr2	47404213	0	0	1	0	C	C	T	C	targeted
chr2	47407424	0	0	1	0	C	C	A	C	targeted
chr2	47407996	0	0	1	0	A	A	G	A	targeted
chr2	47407997	0	0	1	0	T	T	G	T	targeted
chr2	47409926	0	0	1	0	C	C	T	C	targeted
chr2	47410874	0	0	1	0	C	C	G	C	targeted

chr2	47411656	0	0	1	0	A	A	G	A	targeted
chr2	47412190	0	0	1	0	A	A	G	A	targeted
chr2	47416052	0	0	1	0	C	C	G	C	targeted
chr2	47418110	0	0	1	0	G	G	T	G	targeted
chr2	47418510	0	0	1	0	G	G	A	G	targeted
chr2	47420699	0	0	1	0	C	C	T	C	targeted
chr2	47420838	0	0	1	0	G	G	A	G	targeted
chr2	47424109	0	0	1	0	T	T	C	T	targeted
chr2	47425316	0	0	1	0	C	C	T	C	targeted
chr2	47426360	0	0	1	0	G	G	A	G	targeted
chr2	47426506	0	0	1	0	C	C	T	C	targeted
chr2	47427646	0	0	1	0	G	G	T	G	targeted
chr2	47428327	0	0	1	0	A	A	G	A	targeted
chr2	47429662	0	0	1	0	G	G	A	G	targeted
chr2	47431290	0	0	1	0	T	T	A	T	targeted
chr2	47431335	0	0	1	0	A	A	T	A	targeted
chr2	47431571	0	0	1	0	A	A	G	A	targeted
chr2	47433053	0	0	1	0	C	C	A	C	targeted
chr2	47433529	0	0	1	0	T	T	G	T	targeted

chr2	47433747	0	0	1	0	C	C	G	C	targeted
chr2	47434014	0	0	1	0	C	C	T	C	targeted
chr2	47434396	0	0	1	0	C	C	T	C	targeted
chr2	47434505	0	0	1	0	G	G	A	G	targeted
chr2	47435094	0	0	1	0	T	T	G	T	targeted
chr2	47435095	0	0	1	0	T	T	A	T	targeted
chr2	47435589	0	0	1	0	A	A	G	A	targeted
chr2	47436706	0	0	1	0	G	G	A	G	targeted
chr2	47438558	0	0	1	0	T	T	G	T	targeted
chr2	47439253	0	0	1	0	T	T	C	T	targeted
chr2	47440990	0	0	1	0	G	G	A	G	targeted
chr2	47441830	0	0	1	0	G	G	C	G	targeted
chr2	47443165	0	0	1	0	G	G	C	G	targeted
chr2	47443251	0	0	1	0	G	G	A	G	targeted
chr2	47444426	0	0	1	0	A	A	G	A	targeted
chr2	47444603	0	0	1	0	A	A	C	A	targeted
chr2	47445336	0	0	1	0	T	T	A	T	targeted
chr2	47445430	0	0	1	0	G	G	A	G	targeted
chr2	47446376	0	0	1	0	T	T	C	T	targeted

chr2	47446921	0	0	1	0	G	G	C	G	targeted
chr2	47447696	0	0	1	0	C	C	A	C	targeted
chr2	47447735	0	0	1	0	G	G	T	G	targeted
chr2	47448192	0	0	1	0	G	G	C	G	targeted
chr2	47449694	0	0	1	0	A	A	G	A	targeted
chr2	47450480	0	0	1	0	A	A	G	A	targeted
chr2	47451996	0	0	1	0	G	G	C	G	targeted
chr2	47452502	0	0	1	0	A	A	C	A	targeted
chr2	47454351	0	0	1	0	T	T	C	T	targeted
chr2	47456636	0	0	1	0	A	A	G	A	targeted
chr2	47456719	0	0	1	0	C	C	T	C	targeted
chr2	47458075	0	0	1	0	G	G	C	G	targeted
chr2	47458520	0	0	1	0	G	G	C	G	targeted
chr2	47458756	0	0	1	0	C	C	G	C	targeted
chr2	47459237	0	0	1	0	A	A	G	A	targeted
chr2	47459434	0	0	1	0	G	G	T	G	targeted
chr2	47460510	0	0	1	0	A	A	C	A	targeted
chr2	47461698	0	0	1	0	C	C	G	C	targeted
chr2	47461880	0	0	1	0	C	C	T	C	targeted

chr2	47462078	0	0	1	0	G	G	T	G	targeted
chr2	47462117	0	0	1	0	A	A	G	A	targeted
chr2	47463265	0	0	1	0	T	T	C	T	targeted
chr2	47463269	0	0	1	0	A	A	G	A	targeted
chr2	47463272	0	0	1	0	T	T	C	T	targeted
chr2	47463981	0	0	1	0	G	G	A	G	targeted
chr2	47465142	0	0	1	0	C	C	T	C	targeted
chr2	47465493	0	0	1	0	T	T	C	T	targeted
chr2	47466012	0	0	1	0	T	T	A	T	targeted
chr2	47466567	0	0	1	0	G	G	T	G	targeted
chr2	47466649	0	0	1	0	A	A	T	A	targeted
chr2	47466820	0	0	1	0	G	G	A	G	targeted
chr2	47467113	0	0	1	0	C	C	G	C	targeted
chr2	47467173	0	0	1	0	T	T	C	T	targeted
chr2	47469542	0	0	1	0	G	G	C	G	targeted
chr2	47471169	0	0	1	0	A	A	G	A	targeted
chr2	47471245	0	0	1	0	G	G	A	G	targeted
chr2	47473251	0	0	1	0	G	G	A	G	targeted
chr2	47473818	0	0	1	0	C	C	T	C	targeted

chr2	47474566	0	0	1	0	C	C	T	C	targeted
chr2	47476102	0	0	1	0	A	A	G	A	targeted
chr2	47476151	0	0	1	0	G	G	A	G	targeted
chr2	47476367	0	1	0	0	G	T	G	G	targeted
chr2	47476746	0	0	1	0	G	G	A	G	targeted
chr2	47476845	0	0	1	0	T	T	G	T	targeted
chr2	47476969	0	0	1	0	G	G	C	G	targeted
chr2	47479962	0	0	1	0	C	C	T	C	targeted
chr2	47481376	0	0	1	0	G	G	A	G	targeted
chr2	47481700	0	0	1	0	A	A	G	A	targeted
chr2	47481959	0	0	1	0	G	G	T	G	targeted
chr2	47481978	0	0	1	0	C	C	G	C	targeted
chr2	47482014	0	0	1	0	G	G	A	G	targeted
chr2	47485641	0	0	1	0	A	A	C	A	targeted
chr2	47487280	0	0	1	0	G	G	A	G	targeted
chr2	47487306	0	0	1	0	G	G	C	G	targeted
chr2	47488805	0	0	1	0	C	C	T	C	targeted
chr2	47490046	0	0	1	0	T	T	C	T	targeted
chr2	47490768	0	0	1	0	C	C	A	C	targeted

chr2	47491316	0	0	1	0	C	C	G	C	targeted
chr2	47493506	0	0	1	0	T	T	A	T	targeted
chr2	47495475	0	0	1	0	A	A	G	A	targeted
chr2	47498703	0	0	1	0	C	C	T	C	targeted
chr2	47500131	0	0	1	0	G	G	A	G	targeted
chr2	47502683	0	0	1	0	A	A	G	A	targeted
chr2	47505755	0	0	1	0	C	C	G	C	targeted
chr2	47506312	0	0	1	0	G	G	A	G	targeted
chr2	47506396	0	0	1	0	C	C	T	C	targeted
chr2	47506886	0	0	1	0	C	C	T	C	targeted
chr2	47507661	0	0	1	0	C	C	T	C	targeted
chr2	47508922	0	0	1	0	C	C	G	C	targeted
chr2	47510079	0	0	1	0	A	A	C	A	targeted
chr2	47510259	0	0	1	0	C	C	G	C	targeted
chr2	47511909	0	0	1	0	A	A	G	A	targeted
chr2	47511914	0	0	1	0	G	G	A	G	targeted
chr2	47512412	0	0	1	0	A	A	G	A	targeted
chr2	47513059	0	0	1	0	G	G	T	G	targeted
chr2	47513179	0	0	1	0	A	A	G	A	targeted

chr2	47513427	0	0	1	0	T	T	C	T	targeted
chr2	47513877	0	0	1	0	G	G	C	G	targeted
chr2	47514750	0	0	1	0	C	C	A	C	targeted
chr2	47516288	0	0	1	0	G	G	A	G	targeted
chr2	47518162	0	0	1	0	G	G	A	G	targeted
chr2	47518386	0	0	1	0	A	A	G	A	targeted
chr2	47519368	0	0	1	0	C	C	G	C	targeted
chr2	47520173	0	0	1	0	C	C	G	C	targeted
chr2	47521153	0	0	1	0	A	A	G	A	targeted
chr2	47522007	0	0	1	0	G	G	A	G	targeted
chr2	47523088	0	0	1	0	C	C	T	C	targeted
chr2	47523280	0	0	1	0	C	C	T	C	targeted
chr2	47523585	0	0	1	0	C	C	T	C	targeted
chr2	47523601	0	0	1	0	G	G	A	G	targeted
chr2	47524100	0	0	1	0	T	T	C	T	targeted
chr2	47526321	0	0	1	0	C	C	G	C	targeted
chr2	47526531	0	0	1	0	A	A	G	A	targeted
chr2	47526719	0	0	1	0	C	C	T	C	targeted
chr2	47527306	0	0	1	0	T	T	C	T	targeted

chr2	47528215	0	0	1	0	G	G	T	G	targeted
chr2	47532059	0	0	1	0	C	C	A	C	targeted
chr2	47535077	0	0	1	0	T	T	G	T	targeted
chr2	47535257	0	0	1	0	A	A	G	A	targeted
chr2	47535287	0	0	1	0	C	C	A	C	targeted
chr2	47536136	0	0	1	0	G	G	T	G	targeted
chr2	47536756	0	0	1	0	T	T	C	T	targeted
chr2	47539468	0	0	1	0	G	G	T	G	targeted
chr2	47539696	0	0	1	0	G	G	C	G	targeted
chr2	47539727	0	0	1	0	G	G	A	G	targeted
chr2	47541647	0	0	1	0	C	C	T	C	targeted
chr2	47542710	0	0	1	0	G	G	A	G	targeted
chr2	47544538	0	0	1	0	A	A	G	A	targeted
chr2	47545811	0	0	1	0	T	T	C	T	targeted
chr2	47546401	0	0	1	0	A	A	G	A	targeted
chr2	47546501	0	0	1	0	A	A	G	A	targeted
chr2	47547507	0	0	1	0	T	T	G	T	targeted
chr2	47552014	1	1	0	1	C	C	T	C	targeted
chr2	47552122	1	1	0	1	G	G	C	G	targeted

chr2	47552885	0	1	0	1	C	G	C	G	targeted
chr2	47555035	0	1	0	1	G	A	G	A	targeted
chr2	47555617	0	1	0	1	C	A	C	A	targeted
chr2	47555697	0	1	0	1	G	T	G	T	targeted
chr2	47558121	1	1	0	1	C	C	T	C	targeted
chr2	47560570	1	1	0	1	C	C	G	C	targeted
chr2	47561039	0	1	0	1	G	A	G	A	targeted
chr2	47561099	1	1	0	1	G	G	C	G	targeted
chr2	47562230	0	1	0	1	C	T	C	T	targeted
chr2	47562636	1	1	0	1	G	G	A	G	targeted
chr2	47563472	1	1	0	1	T	T	G	T	targeted
chr2	47563885	0	1	0	1	A	G	A	G	targeted
chr2	47564835	0	1	0	1	C	A	C	A	targeted
chr2	47566078	1	1	0	1	T	T	C	T	targeted
chr2	47567821	1	1	0	1	C	C	T	C	targeted
chr2	47568133	1	1	0	1	A	A	G	A	targeted
chr2	47569088	1	1	0	1	A	A	G	A	targeted
chr2	47570898	1	1	0	1	G	G	A	G	targeted
chr2	47571834	1	1	0	1	C	C	T	C	targeted

chr2	47572354	1	1	0	1	A	A	C	A	targeted
chr2	47572997	1	1	0	1	T	T	C	T	targeted
chr2	47573216	1	1	0	1	T	T	C	T	targeted
chr2	47573438	1	1	0	1	A	A	C	A	targeted
chr2	47573464	1	1	0	1	T	T	A	T	targeted
chr2	47574776	1	1	0	1	C	C	T	C	targeted
chr2	47576256	1	1	0	1	A	A	G	A	targeted
chr2	47576590	1	1	0	1	T	T	G	T	targeted
chr2	47576759	1	1	0	1	G	G	T	G	targeted
chr2	47576807	1	1	0	1	A	A	C	A	targeted
chr2	47577155	1	1	0	1	T	T	C	T	targeted
chr2	47577358	1	1	0	1	A	A	T	A	targeted
chr2	47577449	1	1	0	1	A	A	T	A	targeted
chr2	47577874	1	1	0	1	C	C	G	C	targeted
chr2	47578090	1	1	0	1	T	T	C	T	targeted
chr2	47578662	1	1	0	1	G	G	C	G	targeted
chr2	47580354	1	0	0	0	C	T	T	T	targeted
chr2	47580584	0	1	0	1	A	G	A	G	targeted
chr2	47580921	1	0	0	0	T	C	C	C	targeted

chr2	47582164	1	0	0	0	T	G	G	G	targeted
chr2	47583845	1	1	0	1	T	T	G	T	targeted
chr2	47583939	1	0	0	0	G	T	T	T	targeted
chr2	47584427	0	1	0	1	T	G	T	G	targeted
chr2	47584699	1	1	0	1	T	T	C	T	targeted
chr2	47585718	1	0	0	0	T	C	C	C	targeted
chr2	47586766	1	0	0	0	A	G	G	G	targeted
chr2	47586847	0	1	0	1	T	A	T	A	targeted
chr2	47586996	0	1	0	1	T	G	T	G	targeted
chr2	47588274	0	1	0	1	G	A	G	A	targeted
chr2	47588806	1	0	0	0	T	G	G	G	targeted
chr2	47589532	1	0	0	0	C	A	A	A	targeted
chr2	47590322	1	1	0	1	A	A	C	A	targeted
chr2	47590455	1	0	0	0	T	C	C	C	targeted
chr2	47591016	1	1	0	1	G	G	T	G	targeted
chr2	47591342	0	1	0	1	A	G	A	G	targeted
chr2	47591415	0	1	0	1	C	G	C	G	targeted
chr2	47591489	1	1	0	1	G	G	A	G	targeted
chr2	47592200	1	0	0	0	C	A	A	A	targeted

chr2	47594142	1	0	1	0	A	G	A	G	targeted
chr2	47594640	0	0	1	0	A	A	C	A	targeted
chr2	47594792	0	1	0	1	T	C	T	C	targeted
chr2	47595171	1	0	0	0	G	A	A	A	targeted
chr2	47595485	1	0	0	0	G	T	T	T	targeted
chr2	47596119	0	0	1	0	T	T	C	T	targeted
chr2	47596193	1	0	0	0	A	G	G	G	targeted
chr2	47596240	1	1	0	1	T	T	C	T	targeted
chr2	47596662	1	0	1	0	T	C	T	C	targeted
chr2	47596741	0	1	0	1	G	A	G	A	targeted
chr2	47596810	0	0	1	0	C	C	T	C	targeted
chr2	47596900	1	0	1	0	T	C	T	C	targeted
chr2	47597290	0	0	1	0	T	T	A	T	targeted
chr2	47597320	0	1	0	1	G	A	G	A	targeted
chr2	47597378	0	1	0	1	T	C	T	C	targeted
chr2	47597388	1	1	0	1	A	A	C	A	targeted
chr2	47597727	0	1	0	1	C	T	C	T	targeted
chr2	47597808	0	1	0	1	C	T	C	T	targeted
chr2	47598784	1	0	0	0	A	G	G	G	targeted

chr2	47598877	1	0	1	0	C	T	C	T	targeted
chr2	47599082	1	0	1	0	C	T	C	T	targeted
chr2	47599257	0	1	0	1	A	G	A	G	targeted
chr2	47599298	1	0	1	0	G	A	G	A	targeted
chr2	47599542	1	0	1	0	A	G	A	G	targeted
chr2	47599601	0	1	0	1	G	C	G	C	targeted
chr2	47599691	1	0	1	0	A	G	A	G	targeted
chr2	47600881	1	0	1	0	T	A	T	A	targeted
chr2	47601253	1	0	0	0	C	G	G	G	targeted
chr2	47602091	1	0	1	0	G	A	G	A	targeted
chr2	47602339	1	1	0	1	A	A	G	A	targeted
chr2	47603034	1	1	0	1	G	G	C	G	targeted
chr2	47603369	1	1	0	1	C	C	A	C	targeted
chr2	47603552	0	0	0	1	G	G	G	A	targeted
chr2	47603737	1	1	0	1	C	C	G	C	whole_genome
chr2	47604166	1	0	0	0	T	G	G	G	whole_genome
chr2	47604186	1	1	0	1	A	A	T	A	whole_genome
chr2	47604654	0	1	0	1	C	T	C	T	whole_genome
chr2	47605281	1	1	0	1	G	G	T	G	whole_genome

chr2	47606090	1	0	0	0	G	C	C	C	whole_genome
chr2	47606777	0	1	0	1	A	G	A	G	whole_genome
chr2	47606941	0	1	0	1	G	A	G	A	whole_genome
chr2	47607174	1	0	0	0	C	T	T	T	whole_genome
chr2	47607384	1	0	0	0	G	T	T	T	whole_genome
chr2	47607652	1	0	0	0	G	C	C	C	whole_genome
chr2	47608931	1	1	0	1	C	C	T	C	whole_genome
chr2	47609511	1	1	0	1	G	G	A	G	whole_genome
chr2	47611016	0	1	1	1	G	C	C	C	whole_genome
chr2	47611203	0	1	0	1	C	T	C	T	whole_genome
chr2	47611583	0	0	1	0	G	G	A	G	whole_genome
chr2	47611735	1	0	1	0	A	G	A	G	whole_genome
chr2	47611999	0	0	0	1	A	A	A	C	whole_genome
chr2	47616831	0	1	0	1	G	A	G	A	whole_genome
chr2	47617366	1	0	1	0	G	A	G	A	whole_genome
chr2	47617997	0	1	0	1	A	C	A	C	whole_genome
chr2	47618612	0	0	1	0	A	A	G	A	whole_genome
chr2	47618877	1	0	1	0	G	C	G	C	whole_genome
chr2	47619053	0	1	0	1	C	G	C	G	whole_genome

chr2	47619102	0	1	0	1	G	A	G	A	whole_genome
chr2	47619107	0	1	0	1	G	A	G	A	whole_genome
chr2	47619675	1	0	1	0	A	G	A	G	whole_genome
chr2	47619930	1	0	1	0	G	A	G	A	whole_genome
chr2	47620042	0	1	0	1	T	C	T	C	whole_genome
chr2	47620295	0	1	0	1	G	A	G	A	whole_genome
chr2	47620448	0	1	0	1	G	T	G	T	whole_genome
chr2	47621401	0	1	0	1	T	C	T	C	whole_genome
chr2	47621797	1	0	1	0	A	G	A	G	whole_genome
chr2	47622352	0	0	1	0	C	C	T	C	whole_genome
chr2	47624767	1	0	0	0	T	C	C	C	whole_genome
chr2	47625149	1	0	0	0	C	G	G	G	whole_genome
chr2	47625420	1	0	1	0	G	A	G	A	whole_genome
chr2	47625833	0	0	1	0	G	G	A	G	whole_genome
chr2	47625898	0	0	1	0	A	A	T	A	whole_genome
chr2	47626141	0	0	1	0	C	C	G	C	whole_genome
chr2	47626539	0	1	0	1	G	A	G	A	whole_genome
chr2	47627553	1	1	0	1	T	T	C	T	whole_genome
chr2	47627849	1	1	0	1	G	G	C	G	whole_genome

chr2	47628117	0	1	0	1	A	C	A	C	whole_genome
chr2	47628404	0	1	0	1	C	T	C	T	whole_genome
chr2	47628595	1	0	0	0	A	C	C	C	whole_genome
chr2	47629104	0	1	0	1	A	C	A	C	whole_genome
chr2	47629189	0	1	0	1	T	G	T	G	whole_genome
chr2	47630516	0	1	0	1	T	C	T	C	whole_genome
chr2	47630775	1	0	0	0	G	A	A	A	whole_genome
chr2	47630833	1	0	0	0	C	T	T	T	whole_genome
chr2	47631631	1	1	0	1	C	C	T	C	whole_genome
chr2	47631638	1	0	0	0	G	A	A	A	whole_genome
chr2	47632153	1	0	0	0	T	C	C	C	whole_genome
chr2	47633183	1	1	0	1	T	T	G	T	whole_genome
chr2	47633839	0	1	0	1	C	G	C	G	whole_genome
chr2	47635213	0	1	0	1	C	G	C	G	whole_genome
chr2	47637222	0	1	0	1	T	C	T	C	whole_genome
chr2	47638994	0	1	0	1	C	T	C	T	whole_genome
chr2	47639517	0	1	0	1	C	T	C	T	whole_genome
chr2	47639684	0	1	0	1	C	T	C	T	whole_genome
chr2	47640952	0	1	0	1	T	A	T	A	whole_genome

chr2	47641554	0	1	0	1	A	G	A	G	whole_genome
chr2	47641840	0	1	0	1	A	C	A	C	whole_genome
chr2	47644531	0	1	0	1	G	C	G	C	whole_genome
chr2	47644828	0	1	0	1	T	C	T	C	whole_genome
chr2	47644859	1	0	0	0	T	C	C	C	whole_genome
chr2	47645062	0	1	0	1	A	C	A	C	whole_genome
chr2	47645092	0	1	0	1	C	T	C	T	whole_genome
chr2	47645264	0	1	0	1	C	A	C	A	whole_genome
chr2	47645324	0	1	0	1	C	T	C	T	whole_genome
chr2	47645406	1	0	0	0	A	G	G	G	whole_genome
chr2	47645648	1	1	0	1	C	C	T	C	whole_genome
chr2	47645804	0	1	0	1	T	C	T	C	whole_genome
chr2	47646026	0	1	0	1	C	T	C	T	whole_genome
chr2	47646031	0	1	0	1	A	T	A	T	whole_genome
chr2	47647213	0	1	0	1	T	A	T	A	whole_genome
chr2	47647464	0	1	0	1	G	C	G	C	whole_genome
chr2	47647574	0	1	0	1	C	T	C	T	whole_genome
chr2	47647696	0	1	0	1	A	G	A	G	whole_genome
chr2	47647822	0	1	0	1	T	G	T	G	whole_genome

chr2	47648749	0	1	0	1	C	T	C	T	whole_genome
chr2	47649515	0	1	0	1	T	A	T	A	whole_genome
chr2	47649659	0	1	0	1	G	A	G	A	whole_genome
chr2	47649987	0	1	0	1	A	C	A	C	whole_genome
chr2	47650249	0	1	0	1	A	G	A	G	whole_genome
chr2	47650945	1	1	0	1	C	C	A	C	whole_genome
chr2	47652063	0	1	0	1	C	T	C	T	whole_genome
chr2	47654411	1	1	0	1	A	A	G	A	whole_genome
chr2	47655567	0	1	0	1	C	T	C	T	whole_genome
chr2	47656218	0	1	0	1	T	C	T	C	whole_genome
chr2	47656863	0	1	0	1	G	C	G	C	whole_genome
chr2	47658273	0	1	0	1	A	C	A	C	whole_genome
chr2	47658337	0	1	0	1	G	T	G	T	whole_genome
chr2	47658462	1	1	0	1	G	G	T	G	whole_genome
chr2	47658788	0	1	0	1	G	C	G	C	whole_genome
chr2	47659951	0	1	0	1	G	A	G	A	whole_genome
chr2	47661684	0	1	0	1	T	C	T	C	whole_genome
chr2	47662389	1	1	0	1	C	C	A	C	whole_genome
chr2	47662531	1	0	0	0	G	A	A	A	whole_genome

chr2	47663192	0	1	0	1	T	C	T	C	whole_genome
chr2	47663972	0	0	1	0	T	T	C	T	whole_genome
chr2	47664353	1	0	0	0	T	C	C	C	whole_genome
chr2	47664526	0	0	1	0	G	G	T	G	whole_genome
chr2	47664866	0	1	0	1	G	A	G	A	whole_genome
chr2	47665350	0	1	0	1	G	A	G	A	whole_genome
chr2	47665860	0	1	0	1	G	C	G	C	whole_genome
chr2	47667333	0	1	0	1	A	G	A	G	whole_genome
chr2	47669420	1	1	0	1	G	G	C	G	whole_genome
chr2	47670117	1	1	0	1	C	C	G	C	whole_genome
chr2	47670353	1	1	0	1	C	C	G	C	whole_genome
chr2	47670841	1	1	0	1	C	C	G	C	whole_genome
chr2	47674582	1	1	0	1	G	G	A	G	whole_genome
chr2	47674954	1	1	0	1	G	G	A	G	whole_genome
chr2	47675646	1	1	0	1	A	A	C	A	whole_genome
chr2	47676456	1	1	0	1	C	C	T	C	whole_genome
chr2	47676957	1	1	0	1	T	T	C	T	whole_genome
chr2	47679053	1	1	0	1	G	G	A	G	whole_genome
chr2	47680337	1	1	0	1	G	G	A	G	whole_genome

chr2	47682867	0	0	1	0	C	C	G	C	whole_genome
chr2	47682943	1	1	0	1	T	T	C	T	whole_genome
chr2	47683560	1	0	0	0	A	G	G	G	whole_genome
chr2	47683989	1	0	0	0	C	G	G	G	whole_genome
chr2	47685799	1	0	0	0	G	C	C	C	whole_genome
chr2	47686163	1	0	0	0	C	T	T	T	whole_genome
chr2	47686219	1	0	0	0	C	T	T	T	whole_genome
chr2	47686875	1	0	0	0	C	G	G	G	whole_genome
chr2	47688601	1	0	0	0	G	C	C	C	whole_genome
chr2	47688926	1	0	0	0	T	C	C	C	whole_genome
chr2	47689736	0	1	0	1	C	A	C	A	whole_genome
chr2	47694717	1	0	0	0	T	C	C	C	whole_genome
chr2	47699602	0	1	0	1	C	T	C	T	whole_genome
chr2	47701058	0	0	1	0	A	A	T	A	whole_genome
chr2	47704313	0	1	1	1	G	T	T	T	whole_genome
chr2	47706796	0	1	0	1	C	A	C	A	whole_genome
chr2	47707511	1	0	0	0	T	C	C	C	whole_genome
chr2	47707897	0	1	0	1	G	T	G	T	whole_genome
chr2	47713612	0	1	0	1	G	C	G	C	whole_genome

chr2	47713801	0	1	0	1	G	A	G	A	whole_genome
chr2	47714165	0	1	0	1	G	A	G	A	whole_genome
chr2	47714445	0	1	0	1	A	T	A	T	whole_genome
chr2	47714654	0	1	0	1	G	T	G	T	whole_genome
chr2	47714806	0	1	0	1	C	G	C	G	whole_genome
chr2	47714882	0	1	0	1	C	T	C	T	whole_genome
chr2	47715005	0	1	0	1	C	G	C	G	whole_genome
chr2	47715415	0	1	0	1	G	A	G	A	whole_genome
chr2	47715513	0	1	0	1	C	T	C	T	whole_genome
chr2	47715514	0	1	0	1	A	G	A	G	whole_genome
chr2	47715565	0	1	0	1	C	T	C	T	whole_genome
chr2	47715686	0	1	0	1	A	G	A	G	whole_genome
chr2	47715763	0	1	0	1	A	G	A	G	whole_genome
chr2	47716195	0	1	0	1	G	A	G	A	whole_genome
chr2	47716309	0	1	0	1	G	A	G	A	whole_genome
chr2	47716370	0	1	0	1	T	A	T	A	whole_genome
chr2	47716488	0	1	0	1	C	T	C	T	whole_genome
chr2	47716808	0	1	0	1	A	G	A	G	whole_genome
chr2	47720652	0	1	1	1	C	T	T	T	whole_genome

chr2	47720913	0	1	1	1	C	T	T	T	whole_genome
chr2	47720923	0	1	1	1	T	C	C	C	whole_genome
chr2	47720962	0	1	1	1	C	T	T	T	whole_genome
chr2	47721137	0	1	1	1	G	A	A	A	whole_genome
chr2	47721143	0	1	1	1	T	C	C	C	whole_genome
chr2	47721197	0	1	1	1	G	A	A	A	whole_genome
chr2	47721250	0	1	1	1	A	G	G	G	whole_genome
chr2	47721559	0	1	1	1	A	G	G	G	whole_genome
chr2	47721714	0	1	1	1	G	A	A	A	whole_genome
chr2	47721751	0	1	1	1	T	C	C	C	whole_genome
chr2	47723834	0	1	1	1	C	T	T	T	whole_genome
chr2	47724686	0	1	1	1	T	A	A	A	whole_genome
chr2	47724754	0	1	1	1	T	A	A	A	whole_genome
chr2	47725092	0	1	1	1	T	C	C	C	whole_genome
chr2	47725185	0	0	1	0	G	G	A	G	whole_genome
chr2	47725259	0	1	1	1	C	A	A	A	whole_genome
chr2	47725441	0	1	1	1	A	C	C	C	whole_genome
chr2	47725521	0	1	1	1	C	T	T	T	whole_genome
chr2	47725638	0	1	1	1	C	T	T	T	whole_genome

chr2	47725735	0	1	1	1	A	G	G	G	whole_genome
chr2	47725993	0	1	1	1	A	C	C	C	whole_genome
chr2	47726049	0	1	1	1	A	C	C	C	whole_genome
chr2	47726311	0	1	1	1	A	T	T	T	whole_genome
chr2	47727114	0	1	1	1	G	C	C	C	whole_genome
chr2	47727670	1	0	0	0	T	C	C	C	whole_genome
chr2	47728230	0	1	1	1	A	G	G	G	whole_genome
chr2	47733092	0	1	1	1	G	A	A	A	whole_genome
chr2	47739749	0	0	1	0	T	T	A	T	whole_genome
chr2	47740545	1	0	0	0	G	T	T	T	whole_genome
chr2	47740938	0	1	1	1	G	A	A	A	whole_genome
chr2	47747399	0	1	0	1	T	C	T	C	whole_genome
chr2	47750880	0	1	1	1	C	T	T	T	whole_genome
chr2	47751568	0	1	0	1	T	C	T	C	whole_genome
chr2	47752498	0	1	0	1	C	T	C	T	whole_genome
chr2	47752548	0	1	1	1	A	G	G	G	whole_genome
chr2	47754198	0	0	1	0	C	C	A	C	whole_genome
chr2	47755433	0	0	1	0	G	G	A	G	whole_genome
chr2	47758281	0	1	0	1	C	G	C	G	whole_genome

chr2	47759841	0	1	0	1	A	C	A	C	whole_genome
chr2	47760185	0	1	0	1	C	A	C	A	whole_genome
chr2	47762009	0	1	0	1	C	G	C	G	whole_genome
chr2	47764053	0	1	0	1	A	G	A	G	whole_genome
chr2	47765542	0	1	0	1	T	G	T	G	whole_genome
chr2	47765810	0	1	0	1	T	C	T	C	whole_genome
chr2	47766761	0	1	0	1	C	G	C	G	whole_genome
chr2	47768110	0	1	0	1	A	G	A	G	whole_genome
chr2	47768715	0	1	0	1	A	G	A	G	whole_genome
chr2	47769457	0	1	0	1	C	A	C	A	whole_genome
chr2	47769458	0	0	1	0	G	G	A	G	whole_genome
chr2	47770893	0	1	0	1	G	A	G	A	whole_genome
chr2	47771280	0	1	0	1	T	C	T	C	whole_genome
chr2	47771417	0	1	0	1	T	G	T	G	whole_genome
chr2	47771447	0	1	0	1	C	G	C	G	whole_genome
chr2	47773514	0	1	0	1	C	G	C	G	whole_genome
chr2	47773877	0	1	0	1	A	T	A	T	whole_genome
chr2	47775225	0	1	0	1	T	C	T	C	whole_genome
chr2	47775698	0	1	0	1	G	A	G	A	whole_genome

chr2	47776736	0	1	0	1	G	C	G	C	whole_genome
chr2	47778682	0	1	0	1	A	G	A	G	whole_genome
chr2	47782677	0	1	0	1	T	G	T	G	whole_genome
chr2	47783515	0	1	0	1	C	G	C	G	whole_genome
chr2	47783882	0	1	0	1	C	G	C	G	whole_genome
chr2	47783919	0	1	0	1	G	A	G	A	whole_genome
chr2	47783952	0	1	0	1	G	C	G	C	whole_genome
chr2	47784947	0	1	0	1	C	T	C	T	whole_genome
chr2	47785236	0	1	0	1	G	C	G	C	whole_genome
chr2	47785707	1	0	1	0	T	C	T	C	whole_genome
chr2	47785960	0	1	0	1	A	G	A	G	whole_genome
chr2	47786099	0	0	0	1	G	G	G	C	whole_genome
chr2	47786509	0	1	0	1	G	A	G	A	whole_genome
chr2	47788624	1	1	0	1	C	C	T	C	whole_genome
chr2	47789175	0	1	0	1	T	C	T	C	whole_genome
chr2	47789214	0	1	0	1	G	A	G	A	whole_genome
chr2	47789741	1	0	0	0	C	T	T	T	whole_genome
chr2	47789757	0	1	0	1	G	T	G	T	whole_genome
chr2	47790629	0	1	0	1	C	A	C	A	whole_genome

chr2	47790891	1	0	0	0	G	A	A	A	whole_genome
chr2	47790942	0	1	0	0	A	G	A	A	whole_genome
chr2	47791175	0	1	0	1	T	A	T	A	whole_genome
chr2	47791340	0	1	0	1	G	T	G	T	whole_genome
chr2	47791517	0	1	0	1	T	C	T	C	whole_genome
chr2	47792275	0	1	0	1	G	A	G	A	whole_genome
chr2	47792346	1	0	1	0	C	T	C	T	whole_genome
chr2	47793243	0	1	0	1	T	C	T	C	whole_genome
chr2	47794240	0	1	0	1	G	C	G	C	whole_genome
chr2	47795549	0	1	0	1	T	C	T	C	whole_genome
chr2	47795621	0	1	0	1	A	G	A	G	whole_genome
chr2	47795976	0	1	0	0	T	C	T	T	whole_genome
chr2	47796734	0	1	0	1	T	G	T	G	whole_genome
chr2	47797737	1	0	1	0	T	C	T	C	whole_genome
chr2	47798033	1	0	0	0	T	C	C	C	whole_genome
chr2	47804233	0	1	1	1	C	T	T	T	whole_genome
chr2	47805173	0	1	1	1	G	A	A	A	whole_genome
chr2	47805474	0	1	1	1	G	A	A	A	whole_genome
chr2	47813146	0	0	1	0	A	A	C	A	whole_genome

chr2	47814238	0	1	0	1	A	G	A	G	whole_genome

Supplementary Figures

Cloned wild-type sequence:

CTGTGCGCCTAGGACATATGgtatgtgcaaattgttttttccacaaattcggtttttga-
gagggcacttctcttgctagcacatgtatcgctaatatttttctttcttaagGCATGCATGGTAGAAAATGAATGTGAAGACCCCAGCCAGGAGACTATTACGTTCCTC
TATAAATTCATTAAGGGAGCTTGTCCTAAAAGCTATGGCTTTAATGCAGCAAGGCTT-
GCTAATCTCCCAGAGGAAGTTATTCAAAAGGGACATAGAAAAGCAAGAGAATTTGAGAAGATGAATCAGTCACTACGATTATTTCGgta
actaactaactataatggaattataactaactgaccttaagtttcaaagaaacagtaaaaggggaagggatgatgcactatga

Cloned variant sequence showing 74 bp tandem duplication:

CTGTGCGCCTAGGACATATGgtatgtgcaaattgttttttccacaaattcggtttttga-
gagggcacttctgttgctagcacatgtatcgctaatatttttctttcttaagGCATGCATGGTAGAAAATGAATGTGAAGACCCCAGCCAGGAGACTATTACGTTCCTC
TATAAATTCATTAAGGGAGCTTGTCCTAAAAGCTATGGCTTTAATGCAGCAAGGCTT-
GCTAATCTCCCAGAGGAAGT**TATTCAAAAGGGACATAGAAAAGCAAGAGAATTTGAGAAGATGAATCAGTCACTACGATTATTTC**
Ggtaactaaˇ**TATTCAAAAGGGACATAGAAAAGCAAGAGAATTTGAGAAGATGAATCAGTCACTACGAT-**
TATTTCGgtaactaaˇctaactataatggaattataactaactgaccttaagtttcaaagaaacagtaaaaggggaagggatgatgcactatga

Figure S1. Sequence context of the *MSH6* c.3936_4001+8dup variant. The sequence texts are shown for cloned sequences amplified from the genomic DNA of a carrier of the *MSH6* c.3936_4001+8dup variant. Coding sequences are in upper case font and intronic sequences are in lower case font. Primer binding sites for the PCR-amplified region are underlined, with forward primer located at the end of exon 8 and the reverse primer within intron 9. *Top*: Cloned wild-type sequence from the expected 399 bp PCR product. *Bottom*: Cloned variant sequence from the larger 473 bp PCR product showing the 74 bp tandem-duplicated region in bold font, with the duplication in red and indicted at the start and end by ˇ. The region duplicated sequence is shown in bold font. The duplication contains the final 66 bases of exon 9 followed by the first eight bases of intron 9.

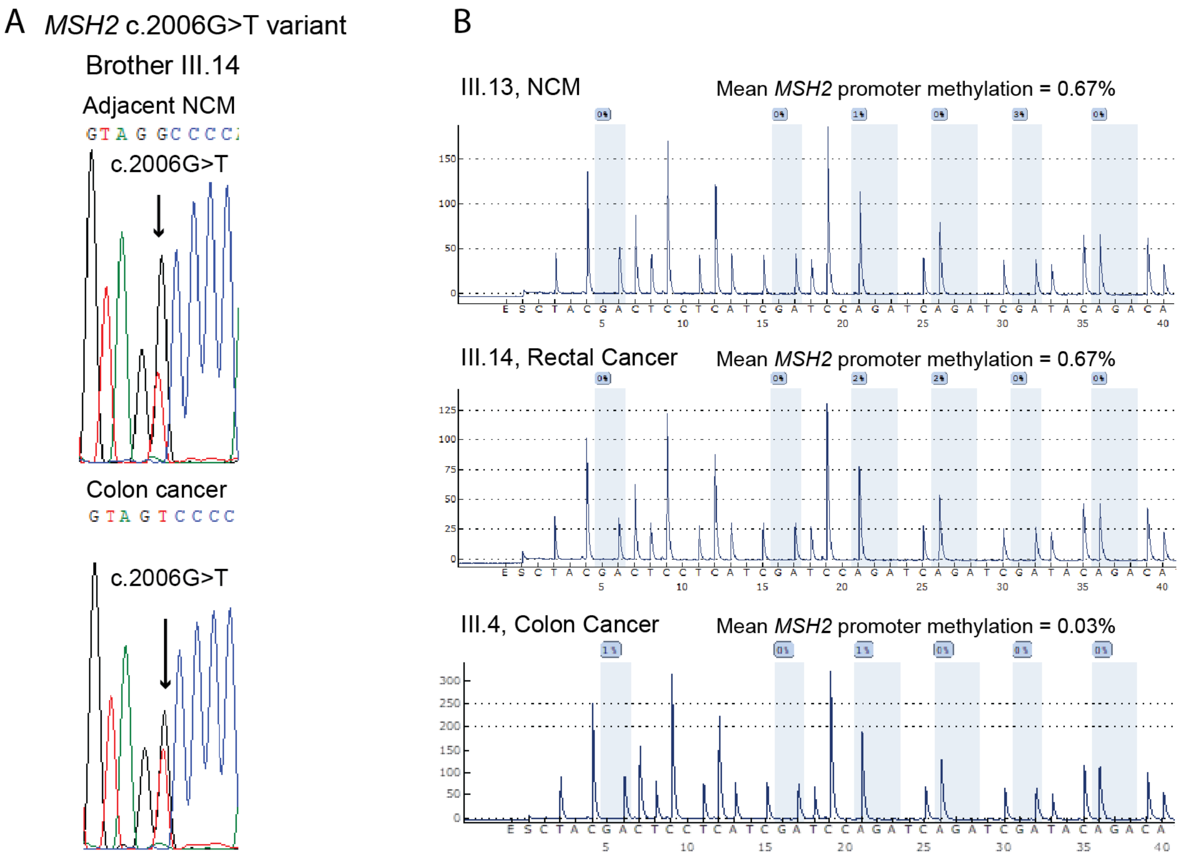

Figure S2. Extended molecular analyses of MSH2-deficient colorectal adenocarcinoma in two brothers who were dual-carriers of both *MSH2* and *MSH6* variants. A: Top, heterozygosity for the *MSH2* c.2006G>T variant in the FFPE-derived adjacent normal colonic mucosa (NCM) from the deceased brother III.14. Bottom, Retention of heterozygosity of both MSH2 c.2006G/T alleles, although the wild-type c.2006G allele was diminished in the colon cancer as compared to the adjacent NCM. B: Representative CpG pyrosequencing traces for a region within the MSH2 CpG island promoter containing six contiguous CpG sites to detect and measure presence of cytosine methylation. Both tumor and NCM samples were negative for *MSH2* promoter methylation.

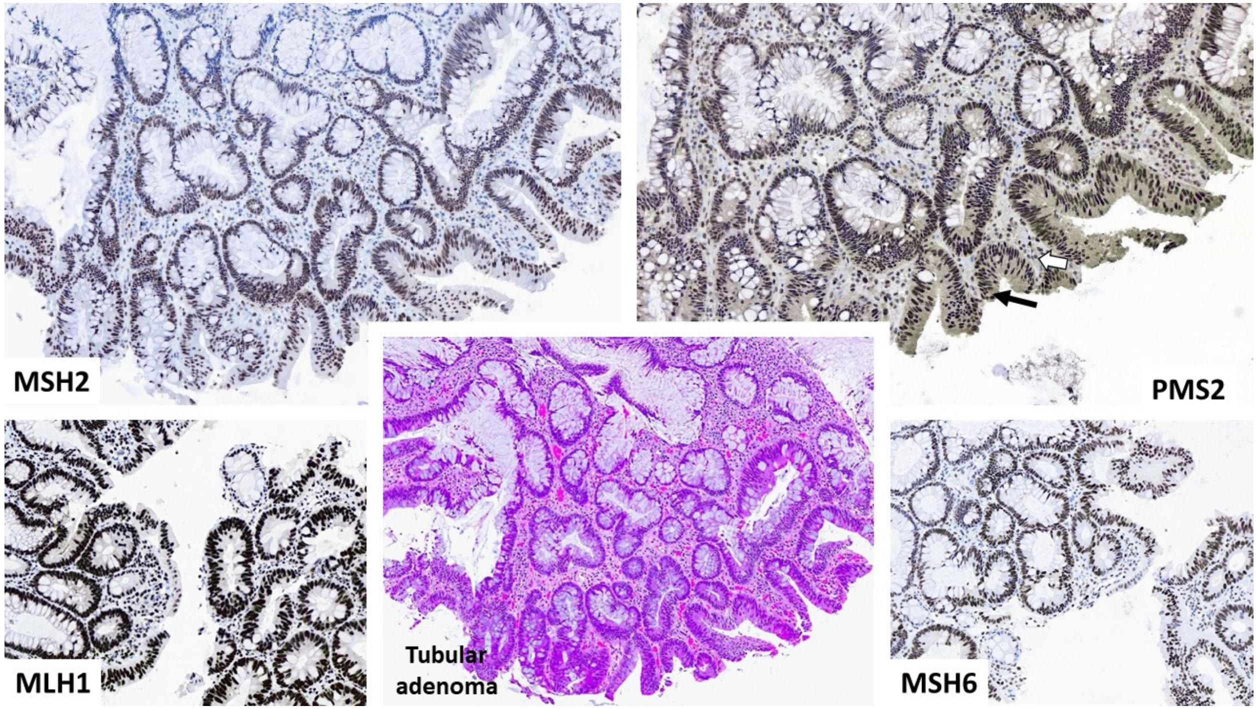

Figure S3. Immunohistochemical staining of the mismatch repair proteins in tubular adenoma. Illustrative composite of one tubular adenoma from a carrier of the *MSH6* variant, showing hematoxylin and eosin stain magnification 10x (center), and intact expression of all four DNA mismatch repair proteins, as labeled. Immunohistochemical stains performed on formalin-fixed, paraffin-embedded colonic adenoma tissue with adjacent non-neoplastic colonic mucosa using antibodies against DNA mismatch repair enzymes MLH1, MSH2, MSH6, and PMS2. Brown staining in nuclei of adenoma cells (black arrow) and in nuclei of lymphocytes as internal controls (white arrow) represents intact expression of MSH6. Each stain was performed on a separate slide. Controls (including internal positive control) were appropriate for each stain.

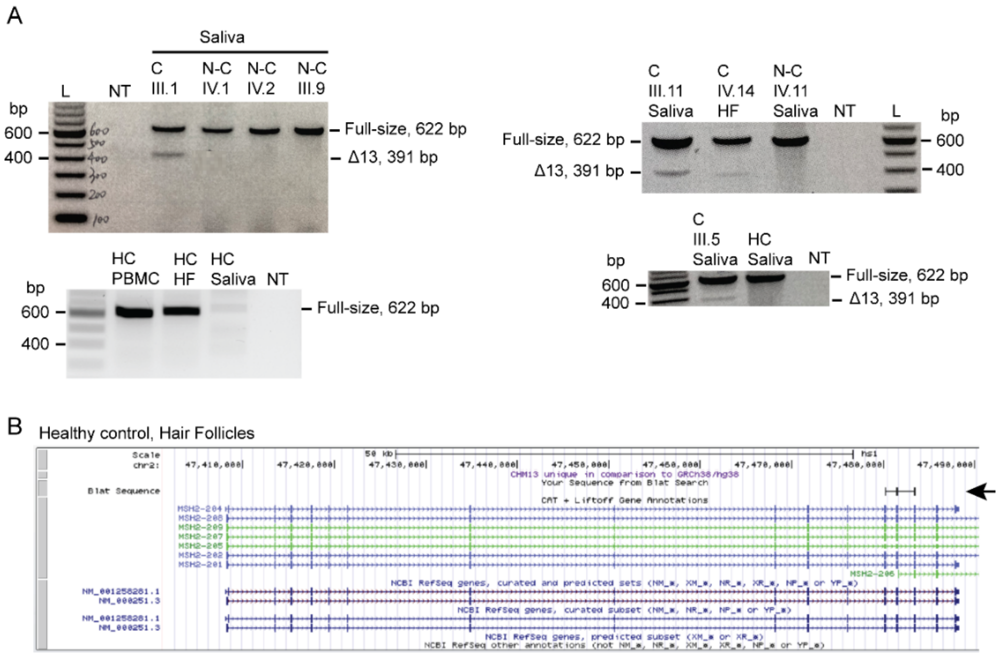

Figure S4. The exon 13-skipped *MSH2* transcript is specific to carriers of the c.2006G>T variant in Family C. A: Scanned photographs of electrophoresis gels after fractionation of *MSH2* exon 12-14 cDNAs in samples from members of Family C who were carriers (C), or non-carriers (N-C), of the *MSH2* c.2006G>T variant and a healthy control (HC) subject. The exon 13-skipped (391 bp) fragment was detected only in carriers. Interestingly, the density of this fragment relative to the full-size fragment was diminished in saliva samples. L, 100 bp ladder. HF, hair follicles; PBMC, peripheral blood mononuclear cells; NT, no template. B: Illustrative example of the alignment of the Sanger sequenced cDNA from the healthy control subject using the UCSC BLAT tool to search the reference human genome (hg38). The “BLAT sequence” interrogated (indicated by a black arrow) showed the amplified cDNA mapped to *MSH2* with inclusion of all three exons 12, 13, and 14. The same results were obtained for HC saliva and PBMCs.

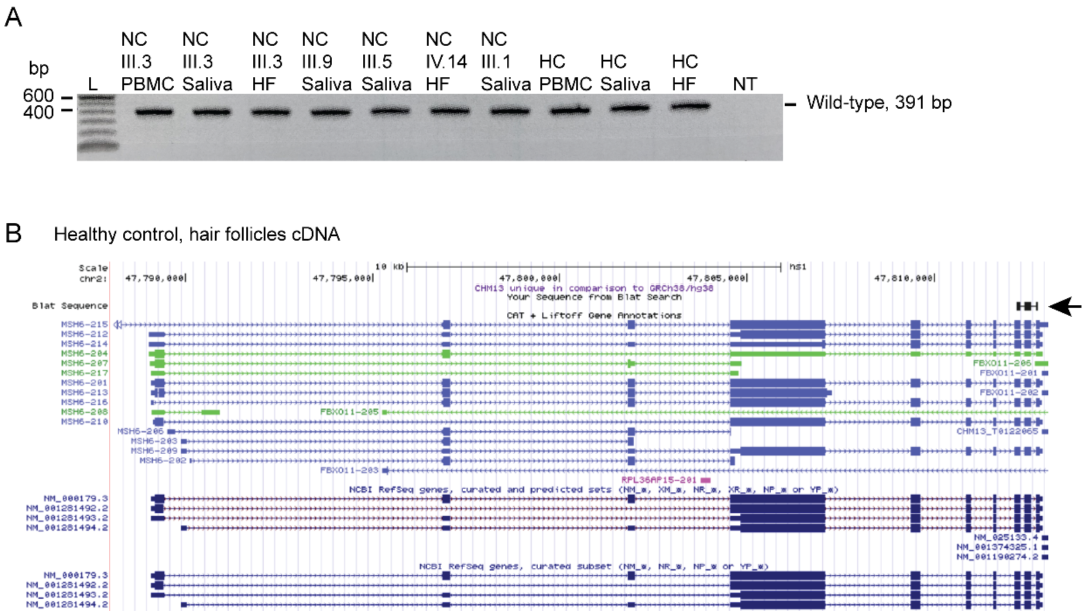

Figure S5. ***MSH6*** **transcripts are wild-type in non-carriers of the** ***MSH6*** **c.3936_4001+8dup (Intronic) variant.** A: Scanned photograph of electrophoresis gels following fragment separation of *MSH6* exon 8-10 cDNAs in various samples from members of Family C who are non-carriers (N-C) of the *MSH6* variant, and a healthy control (HC) subject. Only the normal length (wild-type) transcript fragment was detected in non-carriers in different tissue types. PBMC, peripheral blood mononuclear cells, HF, hair follicles. B: Amplicons were Sanger sequenced and the BLAT tool of the UCSC human genome browser was used to search for matching sequences in the reference human genome (Hg38). An illustrative example is shown in which the cDNA sequence from the healthy control hair follicles was as the "BLAT sequence" (indicated by a black arrow), which mapped to exons 8, 9, and 10 of *MSH6*. The same results were obtained for healthy control saliva and PBMC samples.
